# Arthroscopic Repair of Isolated Subscapularis Tears Show Clinical and Structural Outcome Better for Small Tears Than Larger Tears

**DOI:** 10.1016/j.asmr.2022.04.006

**Published:** 2022-05-28

**Authors:** Hideki Kamijo, Hiroyuki Sugaya, Norimasa Takahashi, Keisuke Matsuki, Morihito Tokai, Yusuke Ueda, Shota Hoshika

**Affiliations:** a Sports Medicine and Joint Center, Funabashi Orthopaedic Hospital, Funabashi, Chiba, Japan; b Tokyo Sports and Orthopaedic Clinic, Toshima, Tokyo, Japan

## Abstract

**Purpose:**

To retrospectively investigate the mid-term outcomes after arthroscopic repair of isolated subscapularis tears with a relatively large number of patients and to compare them by tear size.

**Methods:**

Medical records were reviewed for patients who underwent arthroscopic rotator cuff repair between 2010 and 2017 at our institute. The inclusion criterion was isolated subscapularis tears that underwent arthroscopic rotator cuff repair. The exclusion criteria were (1) previous rotator cuff surgery, (2) lack of imaging studies or clinical evaluation data, (3) neuromuscular diseases, and (4) <2-year follow-up. Range of motion, American Shoulder and Elbow Society score, and bear-hug or belly-press test were assessed pre- and postoperatively. Repair integrity was evaluated with magnetic resonance imaging at postoperative1 year. The clinical and imaging study outcomes were compared between smaller (Lafosse types 1-3) and larger (types 4 and 5) tears.

**Results:**

The subjects included 38 males and 8 females with a mean age of 59 years (range, 25-77 years). The mean follow-up was 36 months (range, 24-96 months). There were 13 type 1, 10 type 2, 12 type 3, 6 type 4, and 5 type 5 shoulders. Postoperative American Shoulder and Elbow Society scores were significantly better in smaller tears than larger tears: 93 ± 8 and 75 ± 14, respectively (*P* = .003). Smaller tears showed better postoperative internal rotation than larger tears (*P* = .004). Significant decrease of positive bear-hug or belly-press test was observed in smaller tears (preoperative, 25; postoperative, 11; *P* < .001), but there was no significant improvement in larger tears (preoperative, 11; postoperative, 9). The retear rate was significantly greater in larger tears (64%) than smaller tears (6%, *P* < .001).

**Conclusions:**

The clinical and structural outcomes after arthroscopic repair of isolated subscapularis tears were better in smaller tears than larger tears with a mid-term follow-up. Larger tears showed high retear rates with poorer improvement in active range of internal rotation and subscapularis strength.

**Level of Evidence:**

Level III, retrospective, comparative study.

Isolated subscapularis tears are relatively rare, accounting for 4% to 5% of all arthroscopic cuff repairs.^1^^,^^2^ As the subscapularis is the dominant internal rotator of the shoulder, the rupture of its tendon can cause pain and shoulder disfunction due to disruption of the transverse force couple.^3^ Since the report by Gerber and Krushell in 1991,^4^ many articles have reported good clinical outcomes after open repair of isolated subscapularis tears.^5^, ^6^, ^7^ Recently, several studies have reported the clinical outcomes of arthroscopic repair of isolated subscapularis tears.^1^, ^2^, ^3^^,^^8^, ^9^, ^10^, ^11^, ^12^, ^13^

Larger subscapularis tears often are associated with fatty degeneration of the muscle,^14^ which has been reported to be a poor prognostic factor in supra- and infraspinatus tears. Wickman et al.^15^ have reported the correlation between tear size and the clinical outcome after arthroscopic repair of supra- and infraspinatus tears. Several previous studies have demonstrated that tear size of the subscapularis tendon was not significantly correlated with the outcomes^3^^,^^7^; however, other studies have shown that large subscapularis tears with advanced fatty degeneration were associated with the high retear rates.^12^^,^^13^ Thus, the relationship between the size of subscapularis tears and surgical outcomes remains unclear. The purpose of this study was to retrospectively investigate the mid-term outcomes after arthroscopic repair of isolated subscapularis tears with a relatively large number of patients and to compare them by tear size. We hypothesized that larger tears would yield poorer outcomes than smaller tears.

## Methods

### Patient Selection

This was a retrospective study that was approved by the institutional review board of our institute. Medical records were reviewed for patients who underwent arthroscopic rotator cuff repair between January 2010 and December 2017 in our institute. The inclusion criterion of this study was isolated subscapularis tear that were arthroscopically repaired. The exclusion criteria were (1) previous rotator cuff surgery, (2) lack of radiographic or clinical evaluation data, (3) neuromuscular diseases, and (4) <2-year follow-up.

### Clinical Evaluation

Patients were clinically examined by one of the senior surgeons (H.S., N.T., K.M., and M.T.) pre- and postoperatively. Active range of motion including flexion, external rotation at the side, and internal rotation was evaluated preoperatively and at the final follow-up. Flexion and external rotation were measured using a goniometer. Internal rotation was assessed as the reachable spinal level with the thumb. Muscle strength of the subscapularis also was assessed with the bear hug^16^ or belly press test^17^ preoperatively and at the final follow-up. The American Shoulder and Elbow Society (ASES) score^18^ was assessed preoperatively and at postoperative 2 years, which demonstrated good reliability in a systematic review.^19^

### Magnetic Resonance Imaging (MRI)

MRI scans were performed preoperatively and at postoperative 1 year using a 1.5-T scanner (Intera; Philips, Amsterdam, the Netherlands) with a phased-array surface coil in all patients. T2-weighted MRI scans were obtained in axial, oblique coronal (parallel to the long axis of the supraspinatus tendon), and oblique sagittal (perpendicular to the long axis of the supraspinatus tendon) planes using a 3.5-mm slice thickness with a 1-mm slice gap. The parameters for T2-weighted images were as follows: repetition time, 4,000 to 5,000 milliseconds; echo time, 100 milliseconds; field of view, 160 × 160 mm; matrix, 384-512 × 720-800. T1-weighted oblique sagittal images also were taken for evaluation of the cuff muscles: repetition time, 400 milliseconds; echo time, 10.5 milliseconds; field of view, 160 × 160 mm; matrix, 400 × 720.

Fatty degeneration of the cuff muscles was evaluated using Goutallier staging modified by Fuchs.^20^^,^^21^ Postoperative repair integrity of the subscapularis was assessed on T2-weighted images using the Sugaya classification, with types 4 and 5 considered as retears.^22^

### Surgical Procedure

**Fig 1:**
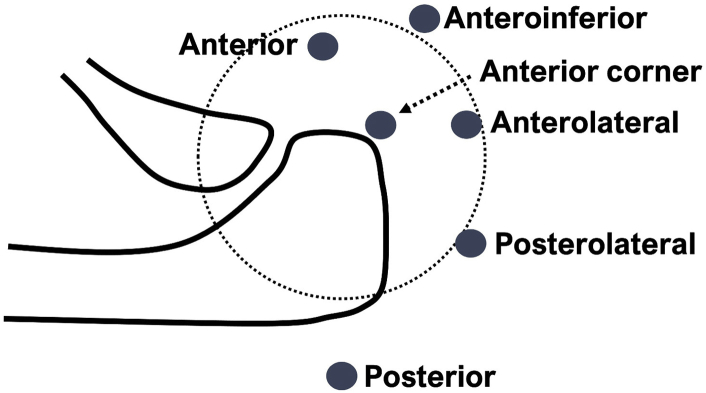
Portal placement (right shoulder, from the top).

All surgeries were performed arthroscopically with the patient under general anesthesia in the beach-chair position. Investigation of the glenohumeral joint was conducted using the standard posterior portal as the viewing portal. After the anterior portal was created in the rotator interval, the scope was introduced into the subacromial space, and the anterolateral and posterolateral portals were created. For subacromial arthroscopy, the posterolateral portal was mainly used as the viewing portal, and the anterior and anterosuperior portals as the working portals (Fig 1).

The integrity of the subscapularis tendon was assessed through both glenohumeral and subacromial views. The transverse humeral ligament was resected to clearly observe the cranial insertion of the scapularis tendon. The size of the subscapularis tendon tear was graded using Lafosse classification.^1^ Tenodesis or tenotomy of the long head of the biceps (LHB) was performed in all patients. The procedures were chosen based on the surgeons’ preference considering patients’ age and sex.^23^ Tenodesis of the LHB was performed just above the cranial border of the pectoris major in the bicipital groove with an interference screw or a suture anchor to create a space for lateral-row anchors for subscapularis repair. When performing LHB tenodesis, an anteroinferior portal was created and used for interference screw or anchor insertion. This portal was also used for subscapularis repair (Fig 1).

**Fig 2:**
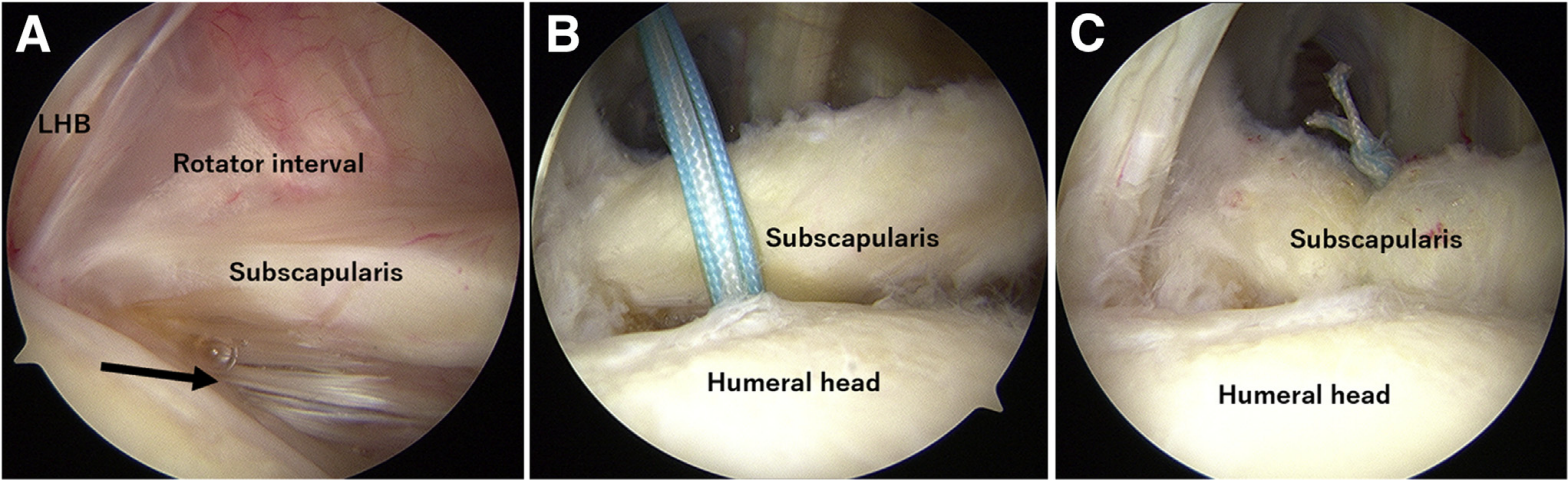
Repair of a Lafosse type 1 tear (left shoulder, intra-articular view from the posterior portal). (A) The arrow indicates the joint side subscapularis tear. (B) A suture anchor loaded with 2 high-strength sutures (HEALICOIL; Smith & Nephew, Andover, MA) was inserted at the most cranial and medial part of the footprint through the anterior portal. (C) The sutures were passed through the tendon using a suture grasper inserted through the anterior portal and tied in a mattress fashion. (LHB, long head of the biceps tendon).

The suture-bridging repair techniques were used in most shoulders, but the single-row repair was applied for some shoulders with a joint-side tear (Lafosse type 1). The single-row repair was performed with the posterior glenohumeral view (Fig 2). A suture anchor loaded with two No. 2 high-strength sutures (HEALICOIL; Smith & Nephew, Andover, MA) was inserted at the most cranial and medial part of the footprint through the anterior portal. The sutures were passed through the tendon using a suture grasper inserted through the anterior portal and tied in a mattress fashion.

The suture-bridging subscapularis repair was performed with the arm at 30° abduction, 45° flexion, and neutral internal/external rotation viewing mainly from the bursa. The rotator interval including the superior glenohumeral ligament was resected, maintaining the fibers that connected the subscapularis and the supraspinatus tendons, which corresponded to the comma sign.^24^ When the subscapularis tear was not clearly visualized, an anterior corner portal was created at the anterior lateral corner of the acromion for the viewing portal (Fig 1) or the comma fibers were released to isolate the subscapularis tear in order to obtain optimal visualization (Fig 1). The released comma fibers were reattached to the supraspinatus after the repair of the subscapularis tendon. After optimal visualization was obtained, extensive release of the tendon was performed for retracted tears, which included the release of the capsule, middle glenohumeral ligament, and subcoracoid adhesion. Resection of the coracohumeral ligament from the coracoid origin was also performed to improve visualization and tendon mobilization.

**Fig 3:**
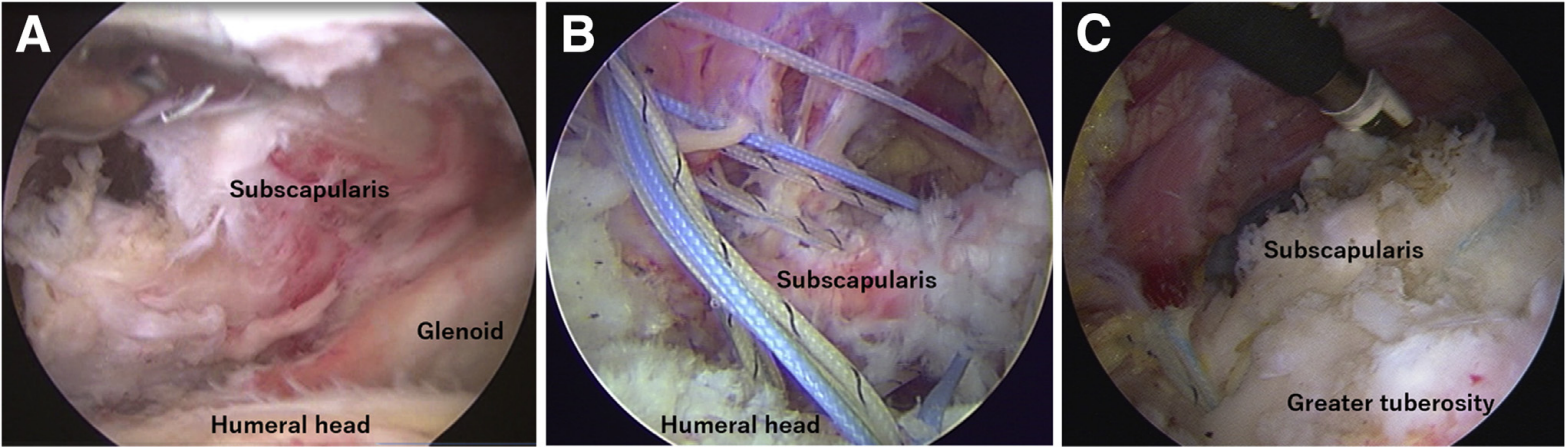
Repair of a large (Lafosse type 4) tear (left shoulder, subacromial view from the posterolateral portal). (A) The subscapularis tendon was completely torn and medially retracted. (B) The sutures of anchors were passed through the tendon. (C) The tendon was repaired using the suture-bridging techniques.

After the tendon release, the lesser tuberosity was decorticated to enhance tendon-to-bone healing. The repair of the subscapularis tendon was performed using the previously reported techniques (Fig 3).^25^^,^^26^ One or two suture anchors loaded with three No. 2 high-strength sutures (HEALIX ADVANCE BR; DePuy Synthes, Raynham, MA) were inserted at the medial border of the footprint through the anterior portal. One or two suture limbs were placed in the tendon at a time using a suture grasper inserted through the anterior portal; 2 suture limbs also were placed in the rotator interval to reduce the comma fibers. After suture passage, suture-bridging was done first using lateral-row anchors (HEALIX ADVANCE KNOTLESS; Depuy Synthes) inserted in the bicipital groove, leaving one pair of suture limbs untied from each anchor. After the completion of suture-bridging, the remaining medial sutures were tied in a mattress fashion.^25^^,^^26^

### PostoperativeTreatment

Shoulders were immobilized using a sling with an abduction pillow (Global Sling; COSMOS, Sapporo, Japan) for 4 weeks. Patients initiated physiotherapy the day after surgery, including relaxation of the shoulder girdle muscles and isometric rotator cuff exercise. After removal of the sling immobilizer, passive and active-assisted shoulder exercises were initiated, and active range motion exercise was started at 6 weeks postoperatively. Patients were allowed light sports activities or jobs 3 months after surgery, and complete return to sports or heavy labor 6 months after surgery, according to their functional recovery.

### Statistical Analysis

All statistical analyses were performed using R (R Foundation for Statistical Computing, Vienna, Austria), and the level of significance was set at *P* < .05. The paired *t*-test and the χ^2^ test were used for comparison of pre- and postoperative values. Mann–Whitney *U* test was used to examine the differences between smaller (Lafosse types 1-3) and larger tear (types 4 and 5) groups. We divided these 2 groups because a recent study using the same repair techniques as this study indicated that type 4 tears had the greater retear rate (3 of 30 shoulders) compared with type 2 and 3 tears (2 of 66 shoulders).^25^

## Results

### Patients

**Fig 4:**
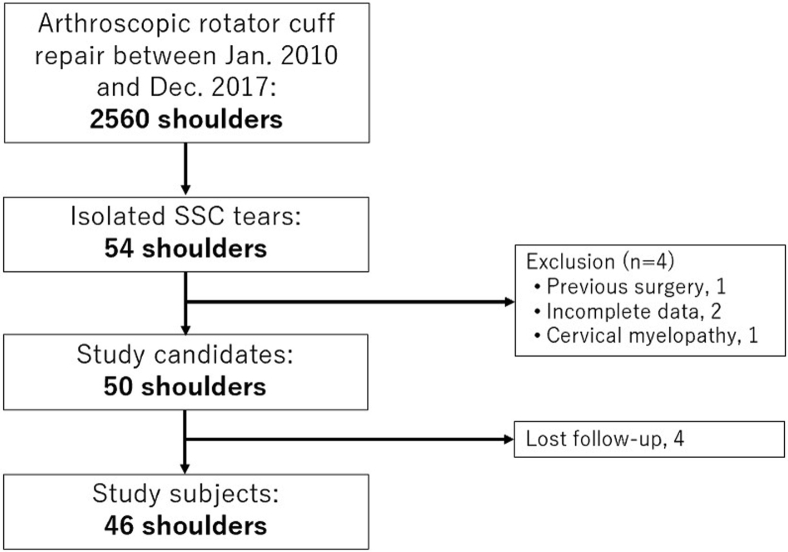
Patient selection.

Arthroscopic repairs of isolated subscapularis tears were performed in 54 (2.1%) shoulders of 2,560 rotator cuff repairs between January 2010 and December 2017 (Fig 4). Four shoulders were excluded from the study: previous surgery, 1 shoulder; incomplete data, 2 shoulders; cervical myelopathy, 1 shoulder. Four patients were not able to be followed up for postoperative 2 years. Thus, 46 shoulders with a minimum 2-year follow-up were included in this study, with the follow-up rate being 92%. They consisted of 38 male and 8 female patients with a mean age of 59 years (range, 25-77 years). The mean follow-up was 36 months (range, 24-96 months). Tear size was classified as type 1 in 13, type 2 in 10, type 3 in 12, type 4 in 6, and type 5 in 5 shoulders. There were 32 traumatic and 14 nontraumatic tears. Five type-1 tears were repaired using the single-row technique, and the remaining 41 shoulders were repaired using the suture-bridging technique. Thirty-five shoulders with type 1-3 tears were assigned to the smaller tear group, and 11 shoulders with type 4 and 5 tears to the larger tear group (Table 1). There were no significant differences in the demographic data between the 2 groups.

**Table 1:** Demographic Data

	Smaller Tears (Types 1-3)	Larger Tears (Types 4 and 5)	*P* Value
Number of shoulders	35	11	
Lafosse classification	Type 1, 13	Type 4, 6 Type 5, 5	
	Type 2, 10		
	Type 3, 12		
Mean age, y (range)	60 (25-77)	57 (38-75)	.5
Sex			
Male	29	9	
Female	6	2	.7
Affected side			
Right	19	6	
Left	16	5	.7
Traumatic tear	24	8	.5
Follow up, mo (range)	37 (24-96)	33 (24-70)	.8
LHB treatment			.3
Tenodesis	26	9	
Tenotomy	6		
Torn	3	2	

LHB, long head of the biceps tendon.

### Clinical Outcomes

ASES scores significantly improved from preoperative to after surgery, respectively, from 56 ± 12 (confidence interval [CI] 52-59) to 89 ± 12 (CI 85-93; *P* < .001). However, postoperative ASES scores were significantly better in smaller (types 1-3) tears than larger (types 4 and 5) tears (Table 2; 93 ± 8 [CI 91-96] and 75 ± 14 [CI 65-86], respectively; *P* = .003). The differences of the ASES scores exceeded the minimal clinically important difference of 6.4.^27^

**Table 2:** Comparison of Clinical Outcomes Between Smaller and Larger Tears

	Smaller Tears (Types 1-3)	Larger Tears (Types 4 and 5)	*P* Value
ASES score			
Preoperative	56 ± 12 (52-60)	55 ± 13 (46-64)	.9
Postoperative	93 ± 8 (91-96)	75 ± 14 (65-86)	**.003**
*P* value	**< .001**	**.006**	
ROM			
Flexion			
Preoperative	160 ± 11 (156-166)	156 ± 23 (141-172]	.5
Postoperative	167 ± 13 (165-170)	166 ± 10 (158-174)	.8
*P* value	.1	.05	
ER			
Preoperative	53 ± 18 (47-59)	64 ± 18 (52-77)	.7
Postoperative	58 ± 14 (52-63)	58 ± 22 (42-75)	.8
*P* value	.2	.5	
IR			
Preoperative	L1 (T12-L2)	L4 (L3-L5)	**.002**
Postoperative	T11 (T10-T12)	L2 (L1-L3)	**.004**
*P* value	**.007**	**.04**	
Bear-hug or belly-press test			
Preoperative	25/35	11/11	**.04**
Postoperative	11/35	9/11	**.003**
*P* value	**< .001**	.1	

NOTE. The ASES scores and ROMs are given as mean ± standard deviation (95% confidence interval). *P* values in bold indicate statistical significance.

ASES, American Shoulder and Elbow Surgeons; ER, external rotation; IR, internal rotation; ROM, range of motion.

Ranges of motion, with the exception of external rotation, significantly improved postoperatively: flexion, 160 ± 16 (CI 155-165) to 167 ± 8 (CI 164-170), *P* = .04; external rotation, 56 ± 18 (CI 50-61) to 58 ± 16 (CI 53-63), *P* = .4; internal rotation, L2 ± 3 (CI L1-L3) to T12 ± 3 (CI T11-T12), *P* < .001. Smaller tears showed better postoperative internal rotation than larger tears (Table 2, *P* = .004), but there were no differences in flexion and external rotation.

The number of shoulders with a positive bear-hug or belly-press test significantly decreased postoperatively (preoperative, 36; postoperative, 20; *P* < .001). A significant decrease of positive tests was observed in smaller tears (preoperative, 25; postoperative, 11; *P* < .001), but there was no significant improvement in larger tears (preoperative, 11; postoperative, 9; *P* = .1).

### MRI Evaluation

Fatty degeneration of the subscapularis muscle did not improve in both smaller and larger tears (Table 3). The larger tears had significantly greater fatty degeneration stages both pre- and postoperative than the smaller tears (*P* < .001 for both).

**Table 3:** MRI Evaluation

	Smaller Tears (Types 1-3)	Larger Tears (Types 4 and 5)	*P* Value
Goutallier stage (stage 1, 2, 3, 4)			
Preoperative	(13, 13, 8, 0)	(0, 4, 2, 5)	**< .001**
Postoperative	(13, 14, 6, 1)	(1, 1, 2, 7)	**< .001**
*P* value	.6	.5	
Retear	2/35 (6%)	7/11 (64%)	**< .001**

NOTE: *P* values in bold indicate statistical significance.

MRI, magnetic resonance imaging.

Retears were found in 2 (6%) shoulders in the smaller tear group and 7 (64%) shoulders in the larger tear group, and the difference was statistically significant (*P* < .001). Of the shoulders with a retear, 2 shoulders required revision surgery. A 72-year-old man with a type 4 tear underwent reverse shoulder arthroplasty at 15 months after arthroscopic subscapularis repair. A 40-year-old man with type 5 tear had revision arthroscopic repair with a fascia lata graft at 10 months after the initial surgery. There were no intra- and postoperative complications.

## Discussion

This study demonstrated that larger isolated subscapularis tears yielded significantly poorer outcomes than smaller tears in terms of ASES score, active range of internal rotation, and the bear-hug/belly-press test, confirming our hypothesis. Fatty degeneration of the subscapularis muscle was significantly more severe in the larger tears than the smaller tears. The retear rate was significantly greater in larger tears than smaller tears.

This study showed significantly poorer clinical outcomes and greater retear rates in shoulders with a larger subscapularis tear than those with a smaller tear. The shoulders with a larger tear also exhibited more severe fatty degeneration of the subscapularis muscle. Yoon et al.^12^ have reported that isolated subscapularis full-thickness tears with stage 3 or 4 fatty degeneration showed higher retear rates (78.6%), although no comparison was made with shoulders with lower grades fatty degeneration in this study. Meshram et al.^13^ have also shown that Lafosse type 4 tears and stage 3 and 4 fatty degeneration were risk factors for retear after subscapularis repair. Thus, more severe fatty degeneration of the subscapularis muscle should be associated with greater retear rates of larger tears, similarly to supra- and infraspinatus tears.^28^ Several studies have reported that subscapularis integrity was important for postoperative clinical outcomes after rotator cuff repair in terms of the transverse force couple.^25^^,^^29^

A significant decrease of positive bear-hug or belly-press tests was seen after surgery in the smaller tears, whereas only 2 patients with larger tears postoperatively demonstrated a negative test. Lafosse et al.^1^ have reported that the lift-off and belly-press tests significantly improved postoperatively in isolated subscapularis tears. Nové-Josserand et al.^2^ also showed that the belly-press test significantly improved postoperativeely in isolated subscapularis tears regardless of arthroscopic or open repair. Shibayama et al.^25^ have indicated that subscapularis tears with greater preoperative Goutallier stages had greater positive rates of postoperative belly-press and bear-hug tests in anterosuperior rotator cuff tears. In this study, more severe fatty degeneration of the subscapularis muscle and the greater retear rate possibly contributed to the high positive rate of the tests in the larger tears. In the smaller tears, the tests significantly improved postoperatively, but no change was seen in fatty degeneration of the subscapularis muscle. This suggested that fatty degeneration did not improve even after a successful repair of the isolated subscapularis tears similar to the findings in supra- and infraspinatus tears.^30^

The results of this study suggest that isolated subscapularis tears should be repaired before tears become larger in size and develop higher-grade fatty degeneration for better clinical outcomes. Previous studies have also reported that early repair of isolated traumatic subscapularis tears yielded good functional outcomes with a low retear rate.^5^^,^^12^ Accordingly, it may be important to diagnose tears as early as possible. More than two-thirds of patients had a traumatic onset in this study, and 78% of patients demonstrated a positive bear-hug or belly-press test. Accurate diagnosis of isolated subscapularis is often difficult, but careful history-taking and physical examination should be helpful and crucial for early diagnosis.

### Limitations

This study had several limitations. First, this is a retrospective study. Second, the number of patients with a larger tear was small. Third, the mean follow-up was only 36 months. The outcomes might be different with a longer-term follow-up study.

## Conclusions

The clinical and structural outcomes after arthroscopic repair of isolated subscapularis tears were better in smaller tears than larger tears with a mid-term follow-up. Larger tears showed high retear rates with poorer improvement in active range of internal rotation and subscapularis strength.

## Supplementary Material

ICMJE author disclosure forms

## References

[1] Lafosse L., Jost B., Reiland Y., Audebert S., Toussaint B., Gobezie R. (2007). Structural integrity and clinical outcomes after arthroscopic repair of isolated subscapularis tears. J Bone Joint Surg Am.

[2] Nové-Josserand L., Hardy M.B., Ogassawara R.L.N., Carrillon Y., Godenèche A. (2012). Clinical and structural results of arthroscopic repair of isolated subscapularis tear. J Bone Joint Surg Am.

[3] Hasler A., Boyce G., Schallberger A., Jost B., Catanzaro S., Gerber C. (2019). Arthroscopic repair of isolated subscapularis tears: Clinical outcome and structural integrity with a minimum follow-up of 4.6 years. J Shoulder Elbow Surg.

[4] Gerber C., Krushell R.J. (1991). Isolated rupture of the tendon of the subscapularis muscle: Clinical features in 16 cases. J Bone Joint Surg Br.

[5] Bartl C., Scheibel M., Magosch P., Lichtenberg S., Habermeyer P. (2011). Open repair of isolated traumatic subscapularis tendon tears. Am J Sports Med.

[6] Petriccioli D., Bertone C., Marchi G., Mujahed I. (2013). Open repair of isolated traumatic subscapularis tendon tears with a synthetic soft tissue reinforcement. Musculoskelet Surg.

[7] Edwards T.B., Walch G., Sirveaux F. (2005). Repair of tears of the subscapularis. J Bone Joint Surg Am.

[8] Katthagen J.C., Vap A.R., Tahal D.S., Horan M.P., Millett P.J. (2017). Arthroscopic repair of isolated partial- and full-thickness upper third subscapularis tendon tears: Minimum 2-year outcomes after single-anchor repair and biceps tenodesis. Arthroscopy.

[9] Rhee Y.G., Lee Y.S., Park Y.B., Kim J.Y., Han K.J., Yoo J.C. (2017). The outcomes and affecting factors after arthroscopic isolated subscapularis tendon repair. J Shoulder Elbow Surg.

[10] Yoon J.S., Kim S.J., Choi Y.R., Kim S.H., Chun Y.M. (2019). Arthroscopic repair of the isolated subscapularis full-thickness tear: Single- versus double-row suture-bridge technique. Am J Sports Med.

[11] Liu Y., Lafosse L., Opsomer G., Villain B., Kempf J.F., Collin P. (2020). Ten-year clinical and magnetic resonance imaging evaluation after repair of isolated subscapularis tears. JSES Int.

[12] Yoon T.H., Kim S.J., Choi Y.R., Keum H.S., Chun Y.M. (2021). Clinical outcomes for isolated subscapularis tears with advanced fatty infiltration: Nonoperative treatment versus arthroscopic single-row repair. Orthop J Sports Med.

[13] Meshram P., Rhee S.M., Park J.H., Oh J.H. (2020). Comparison of functional and radiological outcomes of tears involving the subscapularis: Isolated subscapularis versus combined anterosuperior rotator cuff tears. Orthop J Sports Med.

[14] Nelson G.N., Namdari S., Galatz L., Keener J.D. (2014). Pectoralis major tendon transfer for irreparable subscapularis tears. J Shoulder Elbow Surg.

[15] Wickman J.R., Lau B.C., Scribani M.B., Wittstein J.R. (2020). Single Assessment Numeric Evaluation (SANE) correlates with American Shoulder and Elbow Surgeons score and Western Ontario Rotator Cuff index in patients undergoing arthroscopic rotator cuff repair. J Shoulder Elbow Surg.

[16] Barth J.R., Burkhart S.S., De Beer J.F. (2006). The bear-hug test: A new and sensitive test for diagnosing a subscapularis tear. Arthroscopy.

[17] Gerber C., Hersche O., Farron A. (1996). Isolated rupture of the subscapularis tendon. J Bone Joint Surg Am.

[18] Richards R.R., An K.N., LU Bigliani (1994). A standardized method for the assessment of shoulder function. J Shoulder Elbow Surg.

[19] Roy J.S., MacDermid J.C., Woodhouse L.J. (2009). Measuring shoulder function: A systematic review of four questionnaires. Arthritis Rheum.

[20] Goutallier D., Postel J.M., Benageau J., Lavau J., Voisin M.C. (1994). Fatty muscle degeneration in cuff ruptures: Pre- and postoperative evaluation by CT-scan. Clin Orthop Relat Res.

[21] Fuchs B., Weishaupt D., Zanetti M., Holdler J., Gerber C. (1999). Fatty degeneration of the muscles of the rotator cuff: Assessment by computed tomography versus magnetic resonance imaging. J Shoulder Elbow Surg.

[22] Sugaya H., Maeda K., Matsuki K., Moriishi J. (2007). Repair integrity and functional outcome after arthroscopic double-row rotator cuff repair. J Bone Joint Surg Am.

[23] Kawashima I., Sugaya H., Takahashi N. Biceps tenotomy versus tenodesis in females aged 60 years and older with rotator cuff tears [published online May 31, 2021]. *J Orthop Sci*. 10.1016/j.jos.2021.04.012.

[24] Burkhart S.S., Tehrany A.M. (2002). Arthroscopic subscapularis tendon repair: Technique and preliminary results. Arthroscopy.

[25] Shibayama K., Sugaya H., Matsuki K. (2018). Repair integrity and functional outcomes after arthroscopic suture bridge subscapularis tendon repair. Arthroscopy.

[26] Takeuchi Y., Sugaya H., Takahashi N. (2020). Repair integrity and retear pattern after arthroscopic medial knot-tying after suture-bridge lateral row rotator cuff repair. Am J Sports Med.

[27] Harris J.D., Brand J.C., Cote M.P., Faucett S.C., Dhawan A. (2017). Research pearls: The significance of statistics and perils of pooling. Part 1: Clinical versus statistical significance. Arthroscopy.

[28] Miller B.S., Downie B.K., Kohen R.B. (2011). When do rotator cuff repairs fail? Serial ultrasound examination after arthroscopic repair of large and massive rotator cuff tears. Am J Sports Med.

[29] Ide J., Karasugi T., Okamoto N., Taniwaki T., Oka K., Mizuta H. (2015). Functional and structural comparisons of the arthroscopic knotless double-row suture bridge and single-row repair for anterosuperior rotator cuff tears. J Shoulder Elbow Surg.

[30] Sundararajan S.R., Jha A.K., Ramakanth R., Joseph J.B., Rajasekaran S. (2020). Does change in occupancy ratio and fatty infiltration of the supraspinatus influence functional outcome after single-row rotator cuff repair? A magnetic resonance imaging-based study. J Shoulder Elbow Surg.

